# Effect and individual response to inspiratory muscle training program among instrumentalist musicians

**DOI:** 10.3389/fphys.2024.1522438

**Published:** 2024-12-18

**Authors:** Ana Ibáñez-Pegenaute, María Ortega-Moneo, Robinson Ramírez-Vélez, M. M. Antón

**Affiliations:** ^1^ Department of Health Sciences, Public University of Navarre (UPNA), Pamplona, Spain; ^2^ Navarrabiomed, Hospital Universitario de Navarra (HUN), Universidad Pública de Navarra (UPNA), IdiSNA, Pamplona, Spain; ^3^ CIBER of Frailty and Healthy Aging (CIBERFES), Instituto de Salud Carlos III, Madrid, Spain

**Keywords:** respiratory muscle strength, musicians, breathing exercises, respiratory function tests, responsiveness

## Abstract

In this quasi-experimental before-after trial, we investigated the effects of a high-intensity, low-repetition inspiratory muscle training (HI-LRMT) protocol on respiratory muscle strength in instrumental musicians. In addition, was to estimate the prevalence of “non-responders” (NRs) in terms of muscle force after intervention. Healthy musicians (*n* = 48) were divided into 2 groups: HI-LRMT (*n* = 33) and a control group that did not train (CG, *n* = 15). The intervention training was a high-intensity, low-repetition inspiratory muscle training program using the PowerBreathe^®^ threshold load pressure device, 2 daily sessions of 30 repetitions per session, with a minimum frequency of 5 days per week, for 12 weeks, 5 min per session. The primary outcome measures were maximal inspiratory pressure and expiratory pressure. Change in PImax over 12 weeks for HI-LRMT vs. control was 30.9 (95% CI 25.5–38.3), with the large effect, confirming worthwhile benefits (*ηp* ^2^ = 0.61). There were significant changes in PEmax 37.3 (95% CI 19.3–48.1), with a large effect size (*ηp* ^2^ = 0.33). A third of the participants did not demonstrate improvements in terms of muscle force in instrumental musicians. In conclusion, a 12-week high-intensity, low-repetition inspiratory muscle training program using the PowerBreathe^®^ threshold load pressure device, improved inspiratory and expiratory muscle strength in instrumental musicians.

## 1 Introduction

The strength of the respiratory muscles is fundamental for the performance and health of musicians who play wind instruments, as they require precise and sustained respiratory control. During their performances, these musicians experience significant physical effort, with an average heart rate reaches 60.2% of the maximum theoretical heart rate (HRmax) and can reach up to 76% of HRmax, which corresponds to a moderate work intensity (Iñesta et al., 2008). Respiratory muscle fatigue, defined as the reversible loss of the ability to generate force in the respiratory muscles during repeated contractions, can compromise adequate ventilation (McConnell et al., 1997). This affects both performance and endurance during prolonged performances. To prevent it, a respiratory muscle training (RMT) program can be implemented, designed to improve the strength and function of the inspiratory and expiratory muscles through specific exercises (Sapienza et al., 2002).

RMT has proven effective in various populations, such as patients with COPD and asthma, by improving lung function, quality of life, and exercise tolerance (Geddes et al., 2008). In the sports field, this type of training has also been found to benefit endurance athletes by reducing respiratory muscle fatigue and increasing ventilatory efficiency (Lorca-Santiago et al., 2020). However, there is scarce evidence regarding its application in instrumental musicians (Sapienza et al., 2002; Türk-Espitalier et al., 2024; Yilmaz et al., 2022), which justifies conducting this study.

Although RMT can focus on both types of musculature—inspiratory and expiratory—it is mostly performed on the inspiratory muscles due to its relevance in preventing alveolar hypoventilation and intolerance to physical exercise. Devices like the PowerBreathe_®_ allow adjustable resistance, facilitating high-intensity, low-repetition training similar to that used for other muscle groups. Thus, the main objective of the present study was to evaluate the effect to inspiratory muscle training (IMT) program on respiratory muscle strength in instrumental musicians. In addition, was to estimate the prevalence of “non-responders” (NRs) in terms of muscle force after intervention.

## 2 Methods

Forty-eight musicians come from a band and a professional orchestra, and students and faculty members from a higher conservatory of music, through emails and several talks at the band “La Pamplonesa,” the Navarra Symphony Orchestra (OSN), and the Conservatory of Music of Navarra (CSMN) via email between February 2020 and February 2022. The study complies with the ethical standards of the Declaration of Helsinki and was reviewed and approved by the Ethics Committee of the Universidad Publica de Navarra with code PI-004/20. The inclusion criteria were (i) playing a woodwinds and brass; (ii) playing their main instrument for a minimum of 4 h per week; and (iii) being between 18 and 65 years old. The exclusion criteria were previous initiation of respiratory muscle training; prior diagnosis of chronic lung disease; asthma; and pregnancy.

Musicians were divided into a high-intensity, low-repetition inspiratory muscle training (HI-LRMT) protocol (HI-LRMT, n = 33), and a control group (CG, n = 15) through non-random consecutive sampling. The first 35 recruited musicians formed the HI-LRMT group, while the remaining 15 were assigned to the control group. Training loads for the inspiratory muscles were set based on PImax obtained in the initial assessment. The training protocol consisting of 2 daily sessions of 30 repetitions per session for 5 min, with a minimum frequency of 5 days per week, for 12 weeks. The PowerBreathe^®^ plus series device (POWERbreathe International Ltd. Southam, UK) was used. Resistance is adjusted via a spring-loaded valve according to the musician’s PImax. Depending on the musician’s PImax, a specific model was prescribed, offering different resistances: PwB plus health (17–98 cmH_2_O); PwB plus sport (23–186 cmH_2_O); and PwB competition (29–274 cmH_2_O). For each musician, the device offering the most appropriate resistance ranges was selected based on the work values calculated after measuring PImax. They were instructed to start inspirations from residual volume, performing quick and full vital capacity inspirations against resistance, which varied with training. The intensity began at 30% of their PImax to familiarize themselves with the technique, gradually increasing it to 60%–80% PImax during the first 4 weeks. They were advised to increase it by 10% when they were able to easily complete the 30 repetitions. PImax was measured at 4 and 8 weeks to adjust training loads according to the new PImax. Additionally, a qualitative assessment was conducted at the end of the second week to verify correct training execution and ensure adherence to the program. The modified Borg scale (0–10) was used to monitor and evaluate perceived exertion after each training session. They were recommended to train at an exertion level of 6–7 out of 10 on this scale to avoid excessive fatigue, starting from residual volume and not exceeding 2 min of rest between each inspiration, although preferably consecutively. The prescribed training sessions were performed in an upright and relaxed position, either standing or sitting. The control group continued with their usual routines and were asked to maintain current physical activity levels. All participants baseline testing of outcome measures, including body composition measures, physical activity, and clinical data. PImax/PEmax were measured at baseline and the end of the 12-week program using uniform methodology according to international standards (Calaf, 2004) with DATOSPIR-120 CB spirometer connected to software (PUMA^®^ Version 1.4.2, 21.1 MV, Micro Medical) allowing the evaluators to visualize pressure–time curves. A maximum of ten repetitions of PImax/PEmax were performed, with a minimum of six correct maneuvers, three of which had a variability of <5%. No visual feedback was provided during the tests. We selected the highest among the three reproducible maneuvers. To prevent respiratory muscle fatigue, measurements were taken at intervals of 1 min and 5 min between PImax/PEmax measurements. The equipment was calibrated according to international standards. To effectively interpret PImax/PEmax results, we used the method described by reference equations by Lista-Paz et al. (2023).

Prior to analysis, all data were tested for normality, using the Ryan Joiner test. No data required transformation: raw data values were used in all analyses. The descriptive statistics were given as the mean (95% CI) for the continuous variables and as the percentage (%) for the categorical variables. Baseline comparison of the groups was performed using the independent *t*-tests for continues variables and differences in nominal variables between the HI-LRMT group and the control group were tested with the chi-square test. Comparison of the groups was performed using the repeated measures ANCOVA. The interaction effect between group and time was assessed using repeated measure analysis of covariance (ANCOVA), with the baseline as the covariate. To describe the differences in the related treatments, the effect size between-groups differences were calculated using the partial eta squared (*ηp* ^2^). Measuring muscle force before and after the IMT, expressed in cmH_2_O, helped identify musicians who were unresponsive to the IMT program in terms of muscle force. Participants with improvement under 25 cmH_2_O (considered as the lowest clinically significant difference for muscle force [MCID (Rafferty and Lechtzin, 2024)]) after IMT were classified as non-responders (NRs). As there are no clear definitions for MCID in healthy adults, we assumed that PImax/PImax would improve by 25% following IMT, thus we needed 15 to 25 patients in each group. Statistical significance for these outcomes was accepted at an alpha *α* = 0.05, with study power at *β* = 0.8. Data were stored and analyzed in JASP v 0.17.3.

## 3 Results

Demographic and clinical characteristics of the instrumentalists in the study showed no statistically significant differences between the two groups at the beginning of the study (Table 1).

**TABLE 1:** Characteristics of participants.

Characteristics	Control (*n* = 15)	HI-LRMT (*n* = 33)	*p-V*alue
Sex (male/female) (%)	33/66	60/40	0.074
Age (y)	37.4 (28.1–46.7)	39.1 (35.0–43.2)	0.681
Body mass (kg)	66.4 (59.8–73.1)	67.5 (63.7–71.3)	0.761
Height (m)	1.69 (1.64–1.73)	1.72 (1.69–1.74)	0.221
BMI (kg/m^2^)	23.4 (21.3–25.5)	22.8 (21.9–23.8)	0.516
Body fat (%)	25.0 (20.9–29.2)	20.6 (18.0–23.2)	0.061
Lean tissue mass (kg)	49.5 (44.5–54.5)	53.5 (50.2–56.7)	0.169
Instrument played (woodwind/brasswind) (%)	46/54	52/48	0.293
Weekly instrumental practice (h)	17.5 (14.5–20.4)	17.9 (14.9–21.0)	0.856
Number of years of practice (y)	29.1 (20.7–37.6)	29.4 (25.4–33.3)	0.954
Smoking history (no/yes) (%)	87/13	91/9	0.503
PA levels (MET-min/week)^a^	628.0 (145.7–1110.3)	772.7 (572.5–972.9)	0.493
Absolute PImax (cmH_2_O)	110.1 (101.8–118.3)	102.3 (88.1–116.6)	0.207
PImax (% predicted)^b^	90.8 (80.4–101.3)	94.4 (87.2–101.5)	0.568
Absolute PEmax (cmH_2_O)	122.7 (107.4–138.1)	143.2 (126.8–159.5)	0.120
PEmax (% predicted)^b^	77.4 (68.1–86.7)	80.1 (72.1–88.0)	0.683

Legend: Data are presented as mean (95% CI) unless otherwise stated. PImax: maximal inspiratory pressure; cmH_2_O: centimeters of water; PEmax: maximal expiratory pressure; PImax (% predicted): observed value.

^a^ The Spanish short version of the International Physical Activity Questionnaire (IPAQ) was administered. Data are presented both as continuous variables (in MET/min/week) and as categorical variables (low, moderate and high levels of physical activity defined by < 600, 600–3000, >3000MET/min/week, respectively).

^b^ Reference values were calculated using Lista-Paz´s predictive equations: (1) PImax *(female)* = 61.48 + 0.66 × age +1.55 × BMI, 0.01 × age2; Equation (2) PImax *(male)* = 98.60 + 1.18 × age +0.76 × BMI, 0.02 × age2; Equation (3) PEmax *(female)* = 74.75 + 1.67 × age +1.75 × BMI, 0.02 × age2; Equation (4) PEmax *(male)* = 58.11 + 3.71 × age +2.64 × BMI, 0.04 × age2. They establish cutoff values to indicate respiratory muscle weakness in PImax (62 cmH_2_O for female and 83 cmH_2_O for male) and PEmax (81 cmH_2_O for female and 109 cmH_2_O for male). PImax, and PEmax, values were expressed in both absolute and relative terms (%).

**FIGURE 1:**
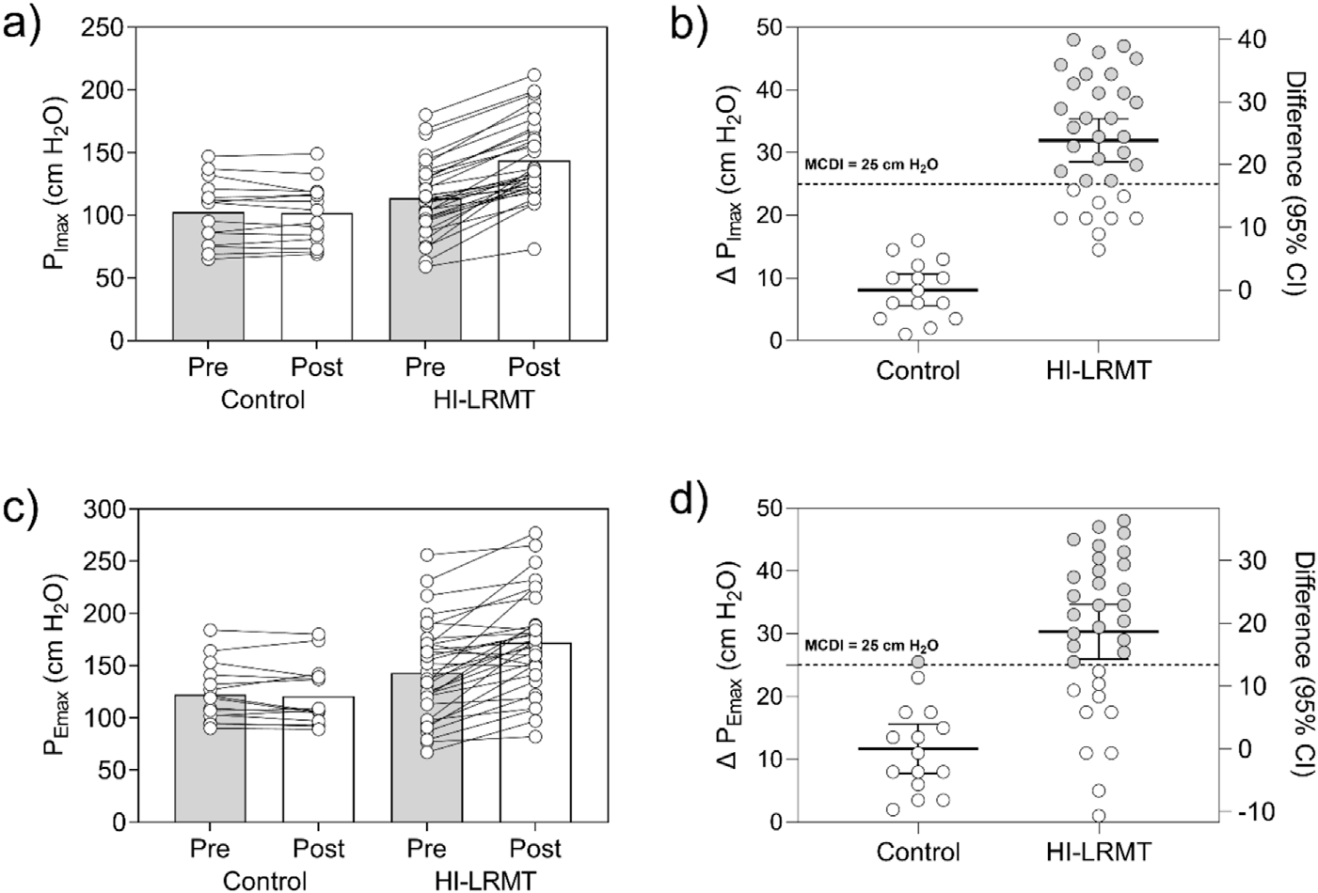
Effects of inspiratory muscle training on maximal inspiratory and expiratory pressure and prevalence of non-responders. Legend: **(A)** Results of the PImax (cmH_2_O) at pre (baseline) and post (12 weeks) according to groups; **(B)** Changes (Δ) and prevalence of responders (gray) and non-responders (white) for PImax (cmH_2_O); **(C)** Results of the PEmax (cmH_2_O) at pre (baseline) and post (12 weeks) according to groups; and **(D)** Changes (Δ) and prevalence of responders (gray) and non-responders (white) for PEmax (cmH_2_O). We use a change of more than 25 cm H_2_O as the threshold to identify change of respiratory muscle strength that does not lie within a normal variance. To determine the effect size of the time × group interaction, the partial eta squared (*ηp* ^2^) was calculated, which was interpreted considering the *ηp* ^2^ values of 0.01, 0.06, and 0.14, which correspond to effect sizes small, moderate, and large, respectively.

Figure 1 presents significant differences (*p* < 0.05) in maximum respiratory pressures between groups, and prevalence of NRs. Change in PImax over 12 weeks for HI-LRMT vs. control was 30.9 (95% CI 25.5–38.3), with the large effect, confirming worthwhile benefits (*ηp* ^2^ = 0.61). There were significant changes in PEmax 37.3 (95% CI 19.3–48.1), with a large effect size (*ηp* ^2^ = 0.33). However, ∼30% of change in PImax (9 out of 33) and change in PEmax (10 out of 33) were classified as NRs in terms of muscle force after IMT (MCID = 25 cmH_2_O). Each musician in the HI-LRMT group kept a diary to record their daily training, intensity, and session observations, which were reviewed during follow-up sessions at weeks 4 and 8 to ensure adherence to the program. The analysis of compliance diaries for the training sessions revealed that participants in the HI-LRMT completed more than 95% of the IMT.

## 4 Discussion

The results of this study prove that a 12-week HI-LRMT with the PowerBreathe^®^ threshold load pressure device significantly increases PImax/PEmax in instrumental musicians. Our results are consistent with previous research, which showed IMT can significantly improve the strength of inspiratory respiratory muscles (Shei, 2018; Craighead et al., 2021). However, there is very little evidence in scientific literature regarding RMT in musicians. Yilmaz et al. (2022) studied the effects of an IMT program at 40% PImax for 4 weeks in vocalist and wind musicians, observing a significant increase in PImax and PEmax in the intervention group. Other studies, such as those by Sapienza et al. (2002), and Türk-Espitalier et al. (2024), conducted expiratory muscle training programs, while Dries et al. (2017) implemented a combined IMT and expiratory muscle training (EMT) program, similarly to Nam et al. (2004) and Ray et al. (2018) in vocalists.

HI-LRMT group obtained significant improvements in PImax and PEmax after 12 weeks of a respiratory muscle training programme; these improvements could be considered clinically relevant, given that they were associated with large effect sizes (≥0.14). In clinical terms, it is crucial that the mean muscle force improvement (+25 cmH_2_O) was significant and aligned with the data in the literature (Rafferty and Lechtzin). Our determination of the MCID value of 25 cmH_2_O for PImax is higher than the value of 18 cmH_2_O established for patients with long-term post-Covid-19 symptoms (Del Corral et al., 2023), and higher than the MCID estimate by Gosselink et al. (2011) of 13 cmH2O in chronic obstructive pulmonary disease. It is important to note that the MCID value for a particular measure can vary depending on the clinical context and decision at hand, the baseline from which the patient starts, and whether they are improving or deteriorating (Del Corral et al., 2023). Thus, the MCID should be judiciously applied to any particular clinical or research context. These elements jointly show that our IMT program was as efficient as in other published studies, and had equivalent mean results (Del Corral et al., 2023).

Following this argument, our study is the first to quantify the individual responses in terms of PImax/PEmax force in relation to the MCID in musicians. Clear evidence also shows that the prevalence of NRs is fully obscured by the mean values, which are influenced by half of the patients improving their muscle force. The amplitude of the improvement in the responders influenced the mean sufficiently that it could induce a significant statistical result. In order to keep this phenomenon in sight, it is thus necessary to express rehabilitation effects in other ways. One complementary approach could be systematic indications of how many people have changes greater than the MCID in order to obtain a more relevant view of the cohort, as well as of the individual and clinical data. The high compliance rate of the RMT program (>95%) indicates that the protocol was well-accepted and feasible to implement. These results are consistent with those obtained by Dries et al. (2017), who reported a 94% adherence rate. This finding is crucial for considering the practical application of IMT in educational and professional contexts for musicians.

This study had some practical implications. For example, the prevalence of NRs reported by the current study may be used to enhance the interpretability and meaningfulness of changes in improvement scores derived from clinical trials that examine the efficacy of interventions designed to improve inspiratory muscle function in musicians. Our findings in terms of the proportion of participants in the experimental group who exceeded the MCID values compared with the proportion of participants in the comparison group, could provide a more clinically relevant method for examining the differences between RMT programs.

In summary, IMT over 12 weeks improved respiratory muscle strength, with third of the participants did not demonstrate improvements in terms of muscle force in instrumental musicians. Thus, there is a clear need for future studies to more accurately express the effects of RMT programs using relevant indicators (e.g., the median improvement or the proportion of patients reaching the MCID). These findings highlight the importance of incorporating respiratory training into musicians’ routines.

## Data Availability

The original contributions presented in the study are included in the article/supplementary material, further inquiries can be directed to the corresponding authors.

## References

[B1] Calaf N. (2004). “Medición de las presiones respiratorias máximas. Sociedad Española de Neumología y Cirugía Torácica,” in Sociedad Española de Neumología y Cirugía Torácica, 134–144.

[B2] Craighead D. H. Heinbockel T. C. Freeberg K. A. Rossman M. J. Jackman R. A. Jankowski L. R. (2021). Time-efficient inspiratory muscle strength training lowers blood pressure and improves endothelial function, NO bioavailability, and oxidative stress in midlife/older adults with above-normal blood pressure. J. Am. Heart Assoc. 10, e020980. Available at: https://doi-org.proxy.bib.uottawa.ca/10.1161/JAHA.121.020980. 34184544 10.1161/JAHA.121.020980 PMC8403283

[B3] Del Corral T. Fabero-Garrido R. Plaza-Manzano G. Fernández-de-Las-Peñas C. Navarro-Santana M. J. López-de-Uralde-Villanueva I. (2023). Minimal clinically important differences in inspiratory muscle function variables after a respiratory muscle training programme in individuals with long-term post-COVID-19 symptoms. J. Clin. Med. 12 (7), 2720. 10.3390/jcm12072720 37048804 PMC10095020

[B4] Dries K. Vincken W. Loeckx J. Schuermans D. Dirckx J. (2017). Effects of a respiratory muscle training program on respiratory function and musical parameters in saxophone players. J. New Music Res. 46 (4), 381–393. 10.1080/09298215.2017.1358751

[B5] Geddes E. L. O’Brien K. Reid W. D. Brooks D. Crowe J. (2008). Inspiratory muscle training in adults with chronic obstructive pulmonary disease: an update of a systematic review. Respir. Med. 102 (12), 1715–1729. 10.1016/j.rmed.2008.07.005 18708282

[B6] Gosselink R. De Vos J. van den Heuvel S. P. Segers J. Decramer M. Kwakkel G. (2011). Impact of inspiratory muscle training in patients with COPD: what is the evidence? Eur. Respir. J. 37 (2), 416–425. 10.1183/09031936.00031810 21282809

[B7] Iñesta C. Terrados N. García D. Pérez J. A. (2008). Heart rate in professional musicians. J. Occup. Med. Toxicol. 3 (1), 16. 10.1186/1745-6673-3-16 18655716 PMC2515327

[B8] Lista-Paz A. Langer D. Barral-Fernández M. Quintela-Del-Río A. Gimeno-Santos E. Arbillaga-Etxarri A. (2023). Maximal respiratory pressure reference equations in healthy adults and cut-off points for defining respiratory muscle weakness. Arch. Bronconeumol 59 (12), 813–820. 10.1016/j.arbres.2023.08.016 37839949

[B9] Lorca-Santiago J. Jiménez S. L. Pareja-Galeano H. Lorenzo A. (2020). Inspiratory muscle training in intermittent sports modalities: a systematic review. Int. J. Environ. Res. Public Health 17 (12), 4448. 10.3390/ijerph17124448 32575827 PMC7344680

[B10] McConnell A. K. Caine M. P. Sharpe G. R. (1997). Inspiratory muscle fatigue following running to volitional fatigue: the influence of baseline strength. Int. J. Sports Med. 18 (3), 169–173. 10.1055/s-2007-972614 9187969

[B11] Nam D. H. Lim J. Y. Ahn C. M. Choi H. S. (2004). Specially programmed respiratory muscle training for singers by using respiratory muscle training device (Ultrabreathe). Yonsei Med. J. 45 (5), 810–817. 10.3349/ymj.2004.45.5.810 15515190

[B12] Rafferty G. F. Lechtzin N. (2024). Tests of respiratory muscle strength. Medilib UpToDate. Available at: https://www.uptodate.com/contents/tests-of-respiratory-muscle-strength?search=Tests%20of%20respiratory%20muscle%20strength&source=search_result&selectedTitle=1%7E150&usage_type=default&display_rank=1 .

[B13] Ray C. Trudeau M. D. McCoy S. (2018). Effects of respiratory muscle strength training in classically trained singers. J. Voice 32 (5), 644.e25–644.e34. 10.1016/j.jvoice.2017.08.005 28958873

[B14] Sapienza C. M. Davenport P. W. Martin A. D. (2002). Expiratory muscle training increases pressure support in high school band students. J. Voice 16 (4), 495–501. 10.1016/s0892-1997(02)00125-x 12512637

[B15] Shei R.-J. (2018). Recent advancements in our understanding of the ergogenic effect of respiratory muscle training in healthy humans: a systematic review. J. Strength Cond. Res. 32 (9), 2665–2676. 10.1519/JSC.0000000000002730 29985221 PMC6105530

[B16] Türk-Espitalier A. Bertsch M. Cossette I. (2024). Effect of expiratory muscle strength training on the performance of professional male trumpet players. Med. Probl. Perform. Art. 39 (1), 18–26. 10.21091/mppa.2024.1003 38413827

[B17] Yilmaz C. Bostancı Ö. Bulut S. (2022). Effect of respiratory muscle training on pitch range and sound duration in brass instrument players and singers. J. Voice 36 (1), 76–82. 10.1016/j.jvoice.2020.04.012 32451252

